# Improved Split-GFP Systems for Visualizing Organelle Contact Sites in Yeast and Human Cells

**DOI:** 10.3389/fcell.2020.571388

**Published:** 2020-11-20

**Authors:** Shinya Tashiro, Yuriko Kakimoto, Manatsu Shinmyo, Shintaro Fujimoto, Yasushi Tamura

**Affiliations:** ^1^Faculty of Science, Yamagata University, Yamagata, Japan; ^2^Department of Biochemistry and Molecular Biology, Graduate School of Medical Science, Yamagata University, Yamagata, Japan

**Keywords:** mitochondria, endoplasmic reticulum, organelle contact site, split GFP, yeast, peroxisome, vacuole, lipid droplet

## Abstract

Inter-organelle contact sites have attracted a lot of attention as functionally specialized regions that mediate the exchange of metabolites, including lipids and ions, between distinct organelles. However, studies on inter-organelle contact sites are at an early stage and it remains enigmatic what directly mediates the organelle-organelle interactions and how the number and degree of the contacts are regulated. As a first step to answer these questions, we previously developed split-GFP probes that could visualize and quantify multiple inter-organelle contact sites in the yeast and human cultured cells. However, the split-GFP probes possessed a disadvantage of inducing artificial connections between two different organelle membranes, especially when overexpressed. In the present study, we developed a way to express the split-GFP probes whose expressions remained at low levels, with minimal variations between different yeast cells. Besides, we constructed a HeLa cell line in which the expression of the split-GFP probes could be induced by the addition of doxycycline to minimize the artificial effects. The improved split-GFP systems may be faithful tools to quantify organelle contact sites and screen new factors involved in organelle-organelle tethering in yeast and mammalian cells.

## Introduction

Organelles are critical membrane-bound structures that developed in eukaryotic cells to allow them to efficiently perform multiple chemical reactions within single cells by isolating and concentrating specific enzymes and metabolites. In order to maintain the characteristic organelle functions, the spatial independence of individual organelles must be strictly maintained so that their contents do not mix via the non-specific fusion of different organelle membranes. However, recent studies have shown that distinct organelles directly interact with each other and form functionally specialized regions called the “organellar-contact sites” (Eisenberg-Bord et al., 2016; Murley and Nunnari, 2016; Cohen et al., 2018; Wu et al., 2018; Scorrano et al., 2019; Tamura et al., 2019; Prinz et al., 2020). For example, it has been revealed that the ERMES (ER-Mitochondria Encounter Structure) complex acts as a molecular tether between the mitochondrial outer membrane and the endoplasmic reticulum (ER) membrane and mediates phospholipid transport between these organelles in yeast (Kornmann et al., 2009; Kojima et al., 2016; Kawano et al., 2018; Kornmann, 2020). In addition to the ER-mitochondria contacts, multiple organelle-contact sites as well as tethering factors have been identified in yeast. Although extensive studies have identified a number of factors that tether distinct organelle membranes, it is still unknown how the contact sites are regulated in terms of number and size. Besides, since we usually visualize organelle contact sites by using fluorescent protein fused to known organelle tethering factors in living cells, it is difficult to observe the contact sites in the absence of such organelle tethering factors. Therefore, we previously developed split-GFP probes that could visualize inter-organelle-contact sites as research tools to tackle these problems (Kakimoto et al., 2018). Our studies using the split-GFP probes in yeast suggest that one organelle forms contact sites with various organelles at the same time. We also showed that the split-GFP system was effective in visualizing the contact site between the mitochondria and the ER in HeLa cells. Similar studies using split-GFP were also performed by other research groups and demonstrated its usability for visualization and quantification of inter-organelle contacts (Cieri et al., 2018; Shai et al., 2018; Yang et al., 2018). In fact, a genome-wide screen using the fluorescent signals of split-Venus as an index of the mitochondria-peroxisome interactions successfully identified Fzo1 and Pex34 as the tethering factors between these organelles (Shai et al., 2018). However, since split-GFP hardly dissociates once it gets associated, it has a disadvantage of inducing artificial inter-organellar contacts, especially when overexpressed. Indeed, a previous study reported that the split-GFP probes expressed on peroxisome and the ER could act as an artificial tether and increase their organelle contacts (Bishop et al., 2019).

In this study, we tested different ways to express split-GFP probes to overcome the drawbacks of the original split-GFP systems. Specifically, we found that expressing split-GFP probes from the genome remarkably decreased the variation in the expression levels among cells and maintained a low expression level in yeast. We also constructed a HeLa cell line that was capable of a doxycycline-inducible expression of the split-GFP protein. By adjusting the doxycycline concentration and induction time, we succeeded in determining the appropriate conditions for observing the ER-mitochondria-contact sites in HeLa cells. Our improved split-GFP methods may be powerful tools for discovering novel organelle tethering factors and regulators.

## Materials and Methods

### Plasmids

Plasmids and DNA oligos used in this study have been listed in Tables 1, 2, respectively. To integrate the genes that express the organelle-targeted split-GFP fragments into the genomic DNA of yeast, we tandemly cloned the *GPD* promoter, the gene encoding the split-GFP protein, the *CYC1* terminator, and the hygromycin or clonNAT-resistant marker gene (*hphMX* or *natNT2*) into the pBlueScript SK(-) cloning vector (Agilent Technologies). We first amplified the DNA fragments encoding the *GPD* promoter, Tom71, Ifa38, Dpp1, the first 60 N-terminal residues of Pex3 and GFP1-10, and the *CYC1* terminator, using the plasmids pSFL22, 16, 100, 26, and 28 (Kakimoto et al., 2018), respectively, as templates and a pair of primers YU1390 and YU499. We also amplified the DNA fragments encoding *hphMX* and *natNT2* by PCR using pBS-hphMX and pBS-natNT2 (Kojima et al., 2019), respectively, as templates and the primer pairs YU501/YU503 and YU500/YU502, respectively. We further amplified the complete gene cassettes that encoded the promoter, the split-GFP fusion gene, the terminator, and the drug-resistance gene by an overlap extension PCR using the two DNA fragments previously mentioned as templates and the primer pairs YU1390/YU503 and YU1390/YU502, and inserted the products into the EcoRI/BamHI site of pBlueScript SK(-) using the In-Fusion^®^ HD Cloning Kit, resulting in pYC135, pYC136, pYC137, pYC141, pYC143, and pYC144, respectively.

**TABLE 1:** Plasmids used in this study.

**Name1**	**Name2**	**Source**
pFL8	pRS316-Su9-RFP	Kakimoto et al., 2018
pFL17	pRS316-BipN-mCherry-HDEL	Kakimoto et al., 2018
pFL92	pRS316-mCherry-Vps39	This study
pSFL9	pRS316-GPDp-GFP(1–10)	Kakimoto et al., 2018
pSFL11	pRS316-GPDp-GFP(11)	Kakimoto et al., 2018
pSFL16	pRS316-GPDp-Ifa38-GFP(1–10)	Kakimoto et al., 2018
pSFL22	pRS316-GPDp-Tom71-GFP(1–10)	Kakimoto et al., 2018
pSFL26	pRS316-GPDp-Pex3N-GFP(1–10)	Kakimoto et al., 2018
pSFL28	pRS316-GPDp-Erg6-GFP(1–10)	Kakimoto et al., 2018
pSFL71	pRS314-GPDp-Tom70N-3xFLAG-GFP(11)	Kakimoto et al., 2018
pSFL100	pRS316-GPDp-Dpp1-GFP(1-10)	Kakimoto et al., 2018
pYC135	pBS-GPDp-Ifa38-V5-GFP(11)-CyC1ter-natNT2	This study
pYC136	pBS-GPDp-Erg6-V5-GFP(11)-CyC2ter-natNT2	This study
pYC137	pBS-GPDp-Dpp1-V5-GFP(11)-CyC1ter-natNT2	This study
pYC141	pBS-GPDp-Ifa38-GFP(1–10)-CyC1ter-hphMX	This study
pYC143	pBS-GPDp-Tom71-GFP(1–10)-CyC1ter-hphMX	This study
pYC144	pBS-GPDp-Pex3N-GFP(1–10)-CyC1ter-hphMX	This study
pMM76	pCDNA3.1-Tom70(1–70)-3xFLAG-eGFP	Kakimoto et al., 2018
pMM77	pCDNA3.1-ERj1N(1–200)-V5-eGFP	Kakimoto et al., 2018
pMM78	pcDNA3.1-N-eGFP-V5-Cb5C	This study
pMM83	pCDNA3.1-Tom70(1–70)-3xFLAG-GFP(11)	This study
pMM86	pCDNA3.1-ERj1N(1–200)-V5-GFP(1–10)	This study
pMM186	pTETOne-ERj1N(1–200)-V5-GFP1-10	This study
pMM189	pIRESNeo3-Tom70(1–70)-3xFLAG-GFP11	This study
pMM220	pCDNA3.1-Tom70(1–70)-3xFLAG-tagBFP	This study
pMM243	pcDNA3.1-Su9-tagBFP	This study
pMM350	pcDNA3.1-mCherry-Cb5C	This study
pYU21	pBS-kanMX4	Kojima et al., 2019
pYU36	pFA6a-mCherry-kanMX6	Kakimoto et al., 2018
pYU60	pRS316-ADH1p-MCS-CYC1ter	This study
pYU99	pRS316-ADH1p-mCherry-MCS-CYC1ter	This study
pYU101	pFA6a-mScarlet-KanMX4	Kakimoto et al., 2018
pJW1513	pRS316-pTDH3-Su9-TagBFP	Williams et al., 2014

**TABLE 2:** Oligo DNAs used in this study.

**Name1**	**Sequence**
YU311	AATTGCGGCCGCATGGTGAGCAAGGGCGAGGAGG
YU312	CCCACTAGTCTTGTACAGCTCGTCCATGCCGCC
YU499	CGCCTCGACATCATCTGCCCGGCCGCAAATTAAAGCCTTC
YU500	GAAGGCTTTAATTTGCGGCCGAGCTCGATTACAACAGGTG
YU501	GAAGGCTTTAATTTGCGGCCGGGCAGATGATGTCGAGGCG
YU502	TAGAACTAGTGGATCCCGGGTTAATTAAGGCGCGC
YU503	TAGAACTAGTGGATCCAGTCTTGACGTGCGCAGCT
YU944	CATGCGGCCGCCGGTGGCACTAGTATGAGCAAAGG
YU945	CCCTCTAGATTACTTTTCGTTGGGATCTTTCG
YU946	CATGCGGCCGCCCGATGGAGGGTCTGGTGGCG
YU947	CCCTCTAGATTATGTAATCCCAGCAGCATTT
YU1365	CCCTCGTAAAGAATTCATGACTGCTCCGTGCTCTC
YU1367	GCAGAGATCTGGATCCTTATGTAATCCCAGCAGCA
YU1390	NNNGCTAGCGCCACCATGGGTGGCACTAG
YU1403	TAGATGCACAAGTGAACACTGAACAAGCATACTCTCAACCATTTAGATACCGGATCCCCGGGTTAATTAA
YU1404	CACCTCGTTGTAAGTGACGATGATAACCGAGATGACGGAAATATAGTACAGAATTCGAGCTCGTTTAAAC
YU1414	CTGCGGCCTAGCTAGCGCCACCATGGCCGCGTCCA
YU1481	GCAGAGATCTGGATCCTTACTTTTCGTTGGGATCT
YU1499	TTTGAAAGCGCCATAAGTGCGCGTGTTTGTGCCTTCTGATATGATATCGTGTTGTAAAACGACGGCCAGT
YU1500	CAATTACACTTTTTTTTTTAGATTGTTCGGTACTTAGTCAAGTTTTATTTCACAGGAAACAGCTATGACC
YU1506	CGCCGGAACCGGCTTTTCATATAGAATAGAGAAGCGTTCATGACTAAATGGTTGTAAAACGACGGCCAGT
YU1507	GAGCCATTAGTATCAATTTGCTTACCTGTATTCCTTTACATCCTCCTTTTCACAGGAAACAGCTATGACC
YU1510	TTTTGATTCGGTAATCTCCGAGCAGAAGGAAGAACGAAGGAAGGAGCACAGTTGTAAAACGACGGCCAGT
YU1511	AATTTTTTTTTTTTCGTCATTATAGAAATCATTACGACCGAGATTCCCGGCACAGGAAACAGCTATGACC
YU1512	AATTTCAGAGGTCGCCTGAC
YU1513	TCATGATTTTCTGTTACACC
YU1514	TGGTTTCAGGGTCCATAAAG
YU1515	TACTGTTACTTGGTTCTGGC
YU1724	NNNGCGGCCGCATGAGCGAGCTGATTAAGGAGAACATG
YU1725	NNNTCTAGATTAATTAAGCTTGTGCCCCAGTTTGCTAG
YU1773	NNNGGATCCATGGCCTCAACTCGCGTTCTTG
YU1774	NNNGCGGCCGCcGCTACTGTAGGCTCTCTTCTGGAAGG
YU2055	GTATGTGGCCACGTAGTAAAAATACGAGAGAAGAAAAGCCTACAGAGTTACGGATCCCCGGGTTAATTAA
YU2056	AGGCAGAGAAGATAGGAAAAAGATAGAACAAAAAATTTGTACATAAATATGAATTCGAGCTCGTTTAAAC
YU2500	NNNGCTAGCATGGTGAGCAAGGGCGAGGAG
YU2501	NNNGGATCCCTTGTACAGCTCGTCCATGCCGC
YU2825	NNNGGATCCATGTTAAGAGCTCAAAAGCTACACT
YU2827	NNNgtcgacTTACTTATTATTTAGCTCATTTATA
YU2933	AAAATGTGAATCCAAGGTTTCAAGAAAATAAGATAAAGTGAATAGGAAGGGTTGTAAAACGACGGCCAGT
YU2934	AGAAAATAACAGCTAGGTTTTAAAATTATATAGCGAGAAGTACAATTCTACACAGGAAACAGCTATGACC
YU2938	CTTCATCAGCAACTGTAGGAGGAGAAAGCAGGTATATAACTAGCCGCAATGTTGTAAAACGACGGCCAGT
YU2939	CATATTTCTATCATTCACTTGTTAGTGCATGAGAAGAAGTAATTGCAATACACAGGAAACAGCTATGACC
YU3018	ATCCTATGTAACGGTTGAAACAGATCATAAGCTGGCTTCAACTAATCCAAGTTGTAAAACGACGGCCAGT
YU3019	AAATGTTTGTTTTTTTATGTAGACACTATTTTCAAACTATCTTTGTTAAACACAGGAAACAGCTATGACC
YU3020	AAATGTTTGTTTTTTTATGTAGACACTATTTTCAAACTATCTTTGTTAAACACAGGAAACAGCTATGACC
YU3021	ATCGGAGAGTATGTATTTGTGTAGTTATGTACTTAGATATGTAACTTAATCACAGGAAACAGCTATGACC

To express split-GFP fusion proteins on the ER membrane and the mitochondrial outer membrane (MOM) in HeLa cells, we prepared two plasmids, pMM186 and 189, that expressed ERj1N-V5-GFP1-10 and Tom70N-FLAG-GFP11, respectively. To construct pMM186 and 189, we first amplified the GFP1-10 and GFP11 genes from pSFL9 and pSFL11 (Kakimoto et al., 2018) using the primers YU944/945 and YU946/947, digested the product using NotI/XbaI, and ligated them into the NotI/XbaI sites of pMM77 and 76 (Kakimoto et al., 2018), resulting in pMM86 and pMM83, respectively. Next, we amplified the ERj1N-V5-GFP1-10 and Tom70N-FLAG-GFP11 genes from pMM86 and pMM83 by PCR using primer pairs YU1365/1481 and YU1414/1367, respectively. The DNA fragment encoding ERj1N-V5-GFP1-10 was cloned into an EcoRI/BamHI-cut pTETone vector (Takara Bio USA, Inc.) using the In-Fusion^®^ HD Cloning Kit, resulting in pMM186. The DNA fragment encoding Tom70N-FLAG-GFP11 was digested with NheI/BamHI and ligated into the NheI/BamHI-cut pIRESNeo3 vector (Takara Bio USA, Inc.), resulting in pMM189.

To express mCherry-Vps39, we first prepared yeast expression vectors with the *ADH1* promoter and the *CYC1* terminator in *CEN*-plasmids, pRS316, resulting in pYU60. Then, we cloned the mCherry gene amplified by PCR using a pair of primer YU311/312 to the NotI/SpeI site of pYU60, resulting in pYU99. Finally, we cloned the *VPS39* gene amplified by PCR using a pair of primer YU2825/2827 to the BamHI/SalI site of pYU99, resulting in pFL92.

To express Su9-tagBFP protein in HeLa cells, we constructed pMM243 as follows. First, we amplified the tagBFP gene by PCR using pJW1513 (Williams et al., 2014) as the template and a pair of primers YU1724/1725, digested with NotI/XbaI, and then ligated to NotI-XbaI-digested pMM76 (Kakimoto et al., 2018), resulting in pMM220. We amplified the DNA fragment encoding the Su9 presequence by PCR using a pair of primers YU1773/1774, digested with BamHI/NotI and ligated to the BamHI/NotI site of pMM220 to replace the gene encoding N-terminal 70 amino acids of Tom70 and 3xFLAG tag to the gene encoding Su9. pJW1513 was a gift from Jonathan Weissman (Addgene plasmid # 62383^1^; RRID:Addgene_62383).

To express mCherry-Cb5C comprising of mCherry and C-terminal 104–134 residues of cytochrome b5 in HeLa cells, we constructed pMM350 as follows. First, we purchased pMM78, pcDNA3.1-N-eGFP vector in which the tandem genes encoding V5 tag and residues 104–134 of cytochrome b5, were cloned at the BamHI/NotI site from eurofin genomics. We then amplified the gene encoding mCherry by PCR using pMM27 (Kakimoto et al., 2018) as template and a pair of primers YU2500/2501, digested with NheI/BamHI and ligated to the NheI/BamHI-digested pMM78 to replace eGFP to mCherry.

### Yeast Strains and Growth Media

A haploid SEY6210 strain (*MAT*α *leu2-3*,*112 ura3-52 his3-Δ200 trp1-Δ901 suc2-Δ9 lys2-801; GAL*) was used in this study. To chromosomally express the split-GFP proteins in yeast, we transformed the wild-type yeast cells with DNA fragments amplified from pYC135, pYC136, pYC137, pYC141, pYC143, or pYC144 by PCR using the primer pairs YU1506/1507 or YU1510/1511. The genes for GFP1-10 and GFP11 were integrated into the *LEU2* and *URA3* loci, respectively. The transformants were selected on the yeast extract-peptone-dextrose (YPD) medium containing 200 μg/ml hygromycin B or 200 μg/ml clonNAT. The integration of the DNA cassettes into the correct sites was confirmed by PCR using the genomic DNA as template and the primer pairs YU1512/1513 for the *LEU2* locus and YU1514/1515 for the *URA3* locus. For the expression of the split-GFP probes from plasmids, yeast cells were cultivated on SCD-Trp-Ura (0.67% yeast nitrogen base without amino acids, 0.5% casamino acids, 2% glucose, 20 μg/ml each of adenine, L-histidine, and L-methionine, and 30 μg/ml each of L-leucine and L-lysine). For the expression of the split-GFP probes from chromosomes, yeast cells were cultivated in the SCD complete medium (0.67% yeast nitrogen base without amino acids, 0.5% casamino acids, 2% glucose, 20 μg/ml each of adenine, L-histidine, L-methionine, L-tryptophan, and uracil, and 30 μg/ml each of L-leucine and L-lysine). For starvation conditions, SD-N or S-NC medium, which omits ammonium sulfate or both ammonium sulfate and glucose from the SCD complete medium were used. To introduce mScarlet or mCherry-tag at C-terminus of Mmm1, Nvj1 and Sei1, we amplified DNA fragments by PCR using pYU36 or pYU101 as the template and primer pairs YU2055/2056, YU1403/1404, YU2931/2932, respectively, and then introduced them into yeast cells expressing the split-GFP proteins. Similarly, to introduce the *mdm12Δ*, *mdm34Δ*, *sei1Δ*, *ldb16Δ*, and *mdm1Δ* mutations, we amplified the DNA cassettes harboring the *kanMX4* flanked by 50 bp of homologous sequences to the up- and down-stream of the *MDM12*, *MDM34*, *SEI1*, *LDB16*, and *MDM1* genes, respectively by PCR using pYU21 as the template DNA and primer pairs YU3018/3019, YU3020/3021, YU2933/2934, YU2938/2939, YU1499/1500, respectively, and introduced them into yeast cells expressing the split-GFP proteins.

### Cell Culture and Transfection

HeLa cells were maintained at 37°C in the Dulbecco’s Modified Eagle Medium (DMEM) with 10% FBS and antibiotics (100 U/ml penicillin and 100 μg/ml streptomycin). DNA transfection was performed using Lipofectamine 2000 (Invitrogen) according to the manufacturer’s instructions. Briefly, 24 h before transfection, HeLa cells were seeded in 35-mm glass-bottom dishes (Iwaki) with a seeding density of 1.5 × 10^5^ cells in 2 ml DMEM with 10% FBS and incubated at 37°C under 5% CO_2_. Then, the HeLa cells were co-transfected with two plasmids for the expression of the GFP1-10 and the GFP11 fusion proteins (1.25 μg each/35-mm dish) and further incubated for 24 h for the microscopic analysis.

HeLa cells capable of stably expressing Tom70N-FLAG-GFP11 or expressing ERj1N-V5-GFP1-10 by induction were constructed as follows. First, the HeLa cells were transfected with pMM189 (pIRESNeo3/Tom70N-FLAG-GFP11) and selected with 500 μg/ml of G418. The resulting HeLa cells were further transfected with pMM186 (pTETone/ERj1N-V5-GFP1-10) and selected with 300 μg/ml of hygromycin.

### Western Blotting

Whole cells extracts were prepared from logarithmically growing yeast cells as reported previously (Kushnirov, 2000). Proteins were separate by SDS-PAGE and were transferred to PVDF membranes (Immobilon-FL Millipore). After blocking with 1% skim milk in TBS-T buffer (10 mM Tris–HCl pH 7.5, 150 mM NaCl, and 0.05% Tween 20), the membranes were incubated with primary antibodies against V5, GFP, Tim23, Tom40, or Tom70 for 2 h at room temperature or for overnight at 4°C. After washing with TBS-T buffer three times and specific proteins were detected by Cy5-conjugated secondary antibodies, goat anti-rabbit or mouse IgG (H+L) (Thermo Fisher Scientific) and analyzed with Typhoon imager (GE Healthcare).

### FACS Analysis

For FACS analysis using yeast cells, we used Cell Sorter SH800 (Sony). Briefly, logarithmically growing yeast cells were first evaluated by scattered light channels to obtain yeast cells of roughly similar cell size. After removal of dead cells showing high fluorescence of propidium iodide, cells were separated with FITC channel (488-nm laser, 525/50-nm band-pass filter). For each analysis, total 100,000 cells were analyzed at 2,000 cells per second.

### Fluorescence Microscopy

We used an Olympus IX83 microscope with a CSU-X1 confocal unit (Yokogawa), 100×, 1.4 NA and 20×, 0.75 NA objectives (UplanSApo, Olympus), and an EM-CCD camera (Evolve 512; Photometrics) and an cMOS camera (Zyla-4, ANDOR) manipulated by Metamorph software (Molecular Devices). GFP or RFP/mCherry/Mitotracker were excited by 488-nm or 561-nm lasers (OBIS, Coherent), and the emissions were made to pass through a 520/35-nm or 617/73-nm band-pass filter, respectively. The confocal fluorescent sections were collected every 0.2 or 0.4 μm of the yeast cells or HeLa cells, respectively. We used the ImageJ software to generate maximum projection images from the obtained confocal images. For the MitoTracker staining, HeLa cells were incubated with 100 ng/ml of the MitoTracker Red CMXRos (Thermo Fisher Scientific) in an Opti-MEM for 30 min at 37°C in 5% CO_2_. Cells were washed twice using DMEM with 10% FBS and subjected to a microscopic observation.

For immunofluorescence microscopy, 48 h before microscopic observation, HeLa cells were seeded in 35 mm glass-bottom dish (Iwaki, D141410) with a seeding density of 8 × 10^4^ in 0.2 ml DMEM supplemented with 10% FBS and incubated. Then, the medium was exchanged to DMEM supplemented with 10% FBS and doxycycline for the expressions of GFP1-10 fusion protein and further incubated for 4, 6, or 8 h before the fixation. The HeLa cells were fixed with pre-warmed 4% PFA in phosphate buffer for 12 min at room temperature and washed three times with PBS. The cells were permeabilized with 0.5% Triton X100 in PBS for 12 min and washed three times with PBS. After blocking with 3% BSA containing PBS for 1 h, the cells were incubated with 1 μg/ml anti-Tom20 antibodies (SantaCruz, sc-11415) in blocking buffer for 15 h at 4°C. Cells were washed three times with PBS and incubated with 2 μg/ml Goat Anti-Rabbit IgG H&L (Alexa Fluor^®^ 594), (abcam, ab150080) in blocking buffer for 1 h at RT. The HeLa cells were washed with PBS three times and observed under Olympus IX83 microscope with a CSU-X1 confocal unit (Yokogawa), a 100×, 1.4 NA, objective (UplanSApo, Olympus) and an cMOS camera (ZYLA-4, ANDOR).

To observe organelle-contact sites in yeast, yeast cells expressing the split-GFP probes from plasmid DNAs or the genomic DNA, were cultivated in SCD-Trp-Ura or SCD complete medium, respectively, to logarithmic phase. The yeast cells were collected by centrifugation (2,000 *g* for 5 s) at room temperature and immediately observed under the fluorescence microscope.

For live-cell imaging, HeLa cells expressing split GFP were stimulated by 300 ng/ml doxycycline 8 h before imaging. Mitochondria were stained with 25 nM MitoTracker Red CMXRos 30 min before imaging. The images of HeLa cells cultured at 37°C in 5% CO_2_ were captured at a rate of one frame per 4s by BZ-X800 (Keyence, Japan), a 100×, 1.45 NA, objective (Nikon, Japan). GFP and Mitotracker were excited by 495-nm and 565-nm light and the emission was passed through 525/50-nm or 605/70-nm band-pass filter, respectively. Obtained images were analyzed with ImageJ or BZ-X800 Analyzer (Keyence, Japan) software.

## Results

### The Overexpressed Split-GFP Probes Induce Artificial Organelle Interactions

**FIGURE 1:**
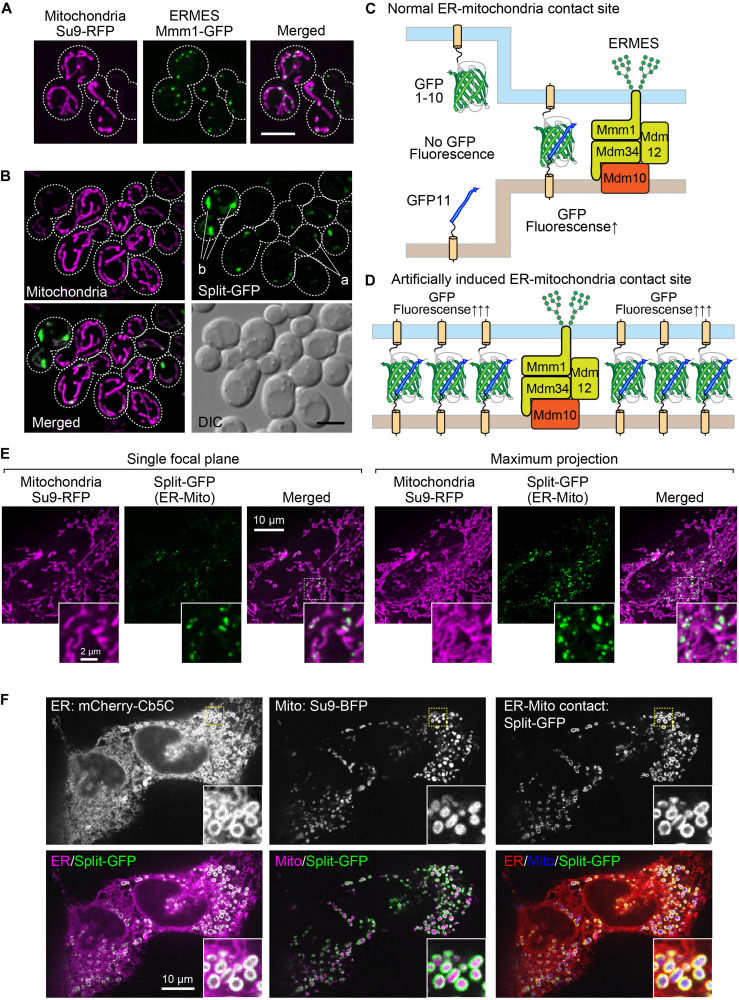
The existing split-GFP systems for visualizing the ER-mitochondria contact sites in yeast and HeLa cells. **(A)** Yeast cells expressing Mmm1-GFP or **(B)** the split-GFP probes on the ER and MOM (Tom70N-GFP1-10 and Ifa38-V5-GFP11) and Su9-RFP were imaged by a confocal fluorescence microscope. Maximum projection images were shown. Scale bars represent 5 μm. “a” indicates GFP signals that resemble ERMES dots. “b” shows abnormal GFP signals that are much larger than ERMES dots. **(C)** Schematic diagrams of the ideal situation that the split-GFP probes work at the ER-mitochondria contact sites and **(D)** the artificial condition that overexpressed split-GFP probes induce undesired ER-mitochondria interactions. **(E)** HeLa cells transiently expressing Tom20N-FLAG-GFP1–10 and ERj1N-V5-GFP11 were stained with MitoTracker and imaged by a confocal fluorescence microscope. A single focal plane (left) and maximum projection image (right) were shown. **(F)** HeLa cells transiently expressing Tom70N-FLAG-GFP11, ERj1N-V5-GFP11, mCherry-Cb5C (ER marker), and Su9-BFP (Mitochondria marker), and were imaged by a confocal fluorescence microscope. A single focal plane was shown.

It has been shown that the ER-mitochondria-contact sites in yeast cells can be observed as small discrete foci under a fluorescent microscope when a subunit of the ERMES complex is expressed as a fusion protein with a fluorescent protein like GFP (Kornmann et al., 2009). For example, when we expressed Mmm1, which is an ER-resident subunit of the ERMES complex, as a GFP-fusion protein, we could observe the “ERMES dots” that represented the ER-mitochondria-contact sites (Figure 1A). We previously assessed the usability of the split-GFP proteins as a fluorescent probe that visualized the organelle-contact sites by using the ERMES dots as references. The split-GFP proteins expressed on the ER and the mitochondrial outer membrane (MOM) mostly showed dot-like signals like the ERMES dots (Figure 1B, a), indicating that the split-GFP proteins worked as a fluorescent probe for the ER-mitochondria-contact sites (Figure 1C). However, we also noticed that the reconstituted split-GFP proteins resulted in some large foci or elongated tubular signals, which were never observed for the ERMES dots, albeit only a minor portion of the total (∼10% of the total signals) (Figure 1B, b). These observations indicate that the split-GFP probes have a disadvantage of inducing artificial organelle-organelle interactions, which in turn affects the organelle morphologies and functions (Figure 1D). Additionally, the GFP signals varied widely among different cells, probably due to the fluctuation of the expression levels of the split-GFP probes, which also makes it difficult to precisely quantify the organelle-organelle interactions.

We previously confirmed that the split-GFP proteins could also visualize the ER-mitochondria-contact sites in human cultured cells. Similar to the ERMES dots, the split-GFP proteins expressed on the ER and the MOM in HeLa cells resulted in punctate signals on the mitochondria (Figure 1E; Kakimoto et al., 2018). This observation indicates that the organelle-targeted split-GFP proteins work as a probe that allows for the visualization of the organelle-contact sites not only in yeasts but also in human cultured cells. However, the overexpressed split-GFP proteins drastically altered the tubular shape of the mitochondria and the ER. We transiently expressed mCherry-Cb5C and Su9-BFP, which were ER and mitochondria marker proteins, respectively, and the split-GFP probes on the ER and MOM at the same time to monitor the organelle morphologies together with their contact sites. We noticed that the overexpressed split-GFP probes deformed both the ER and mitochondria from tubular to vesicular structures, which were hardly seen in normal cells. Besides, we noticed that the vesiculated ball-like mitochondria were completely enclosed by the highly curved ER membranes (Figure 1F). This clearly indicates that the split-GFP proteins affect the structure and function of organelles by inducing organelle-organelle interactions.

### PCR Template Plasmids for the Expression of Split-GFP Probes From the Chromosomal DNA

**FIGURE 2:**
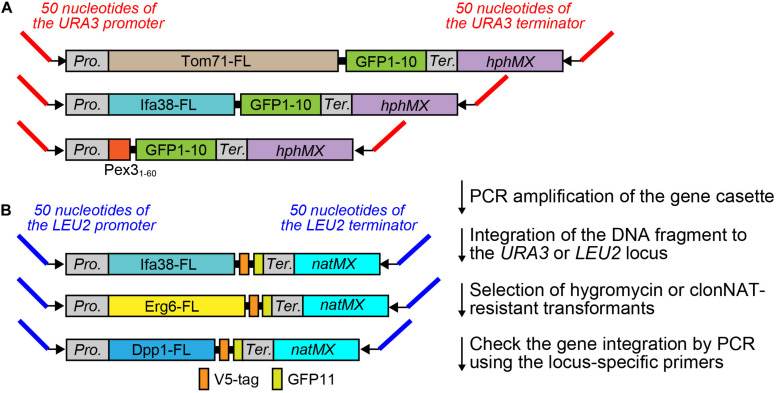
PCR template modules for split-GFP gene integration. **(A)** PCR template modules for GFP1-10 and **(B)** GFP11-fusion genes were shown. Pro. And Ter. indicate the *GPD* promoter and *CYC1* terminator, respectively. FL means full length.

A possible way to overcome the disadvantage of the existing method and for a more precise quantification of the interactions between different organelles using the split-GFP probes in yeast is to modulate the expression levels of the split-GFP probes as well as to lessen their variation among yeast cells. For these purposes, we decided to express the split-GFP probes from the chromosome instead of expression from a plasmid. First, we constructed various gene cassettes that tandemly combined the gene that expressed the split-GFP probe and the drug-resistant gene *hphMX* or *natMX* into the pBlueScript vector (Figures 2A,B). Then, we amplified the gene cassettes from the plasmids by PCR using appropriate primer pairs, as shown in Figure 2 and Table 1. Finally, the purified DNA cassette was transformed into yeast cells by a regular lithium acetate method (Gietz and Schiestl, 2007). In this study, we integrated the GFP1-10 and GFP11 fusion genes into the *URA3* and *LEU2* genes, respectively, which are commonly used as auxotrophic marker genes in yeast (Figure 2).

### Chromosomal Gene Integration Enables Low-Level Expression of the Split-GFP Probes in Yeast

**FIGURE 3:**
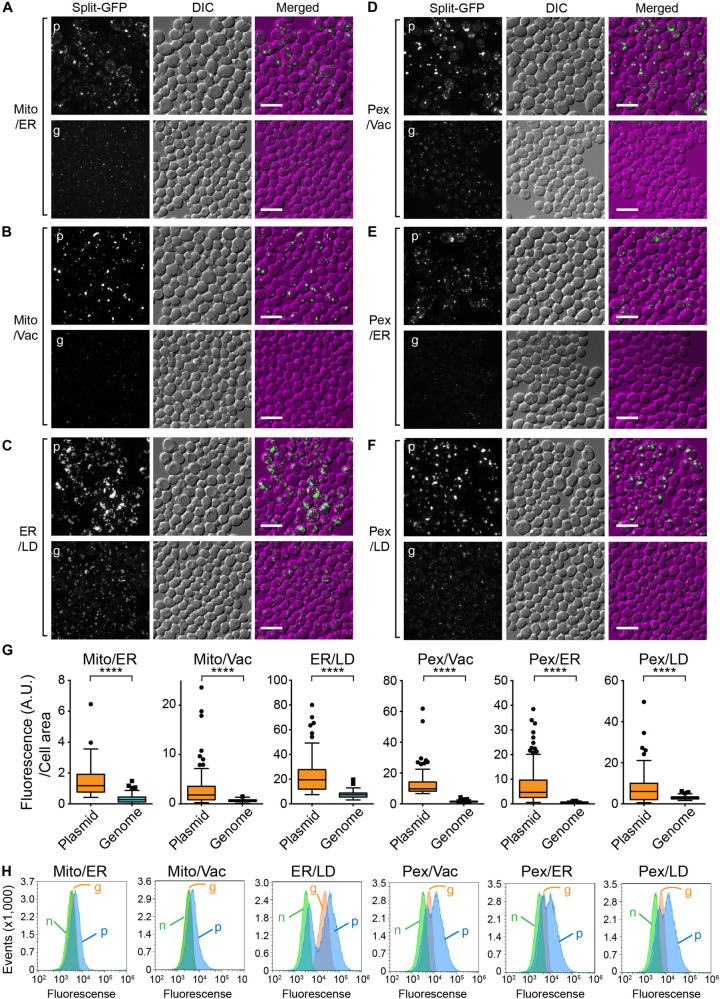
Comparison between chromosomal and plasmid expressions of the split-GFP probes. Yeast cells expressing the split-GFP probes shown in Figure 2 on **(A)** the MOM and the ER, **(B)** the MOM and vacuole, **(C)** the ER and LDs, **(D)** peroxisome and vacuole, **(E)** peroxisome and the ER, **(F)** peroxisome and LD were imaged by a fluorescence confocal microscope. Maximum projection images were shown. Scale bars represent 5 μm. p and g indicate the plasmid- and genome-based expressions of the split-GFP proteins, respectively. **(G)** Box and whisker plots showing the distribution of the fluorescence intensity of GFP signals normalized by the cell area. Over 100 cells were counted for each pair; ****p < 0.0001. **(H)** Logarithmically growing yeast cells expressing the split-GFP probes were subjected to FACS using GFP signal as an index. n, g, and p represent no expression, genome-, and plasmid-based expression of the split-GFP probes, respectively.

We next tested whether integration of the split-GFP genes into the chromosome resulted in lower expression levels of the split-GFP probes and/or smaller variations in their levels among yeast cells. To this end, we selected the following six organelle pairs: mitochondria-ER (Figure 3A), mitochondria-vacuole (Figure 3B), ER-lipid droplets (LDs) (Figure 3C), peroxisome-vacuole, (Figure 3D) peroxisome-ER (Figure 3E), and peroxisome-LDs (Figure 3F). We then compared the reconstituted GFP signals observed at the organelle-contact sites when the split-GFP probes were chromosomally expressed with those obtained when they were expressed from plasmids. As we reported previously, split-GFP probes expressed from plasmids resulted in clear GFP signals between all the organelle pairs tested, although the signal intensities varied widely among different cells, probably due to different expression levels (Kakimoto et al., 2018). Although these results suggest the existence of organelle-contact sites between these organelle pairs, the strong GFP signals probably indicate the artificially induced tethering between these organelles (Figure 1D).

We found that in contrast to the plasmid-expressed split-GFP probes, the chromosomally expressed split-GFP probes exhibited uniform and small punctate GFP signals which were similar to the ERMES dots, indicative of the authenticity of the organelle-contact sites. Quantification of the total GFP intensities normalized to the cell area clearly showed low variations in the GFP signals (Figure 3G). We further confirmed these results by fluorescence-activated cell sorting (FACS). As shown in Figure 3H, we could separate populations of yeast cells with or without the plasmid- or genome-based expression of split-GFP probes. Yeast cells expressing the split-GFP probes from the plasmids showed higher fluorescent signals (Figure 3H, p) than those expressing them from the genomic DNA (Figure 3H, g) and those without the expression (Figure 3H, n). Similar to the result of microscopy-based analyses, the plasmid-based expressions caused large variabilities in the GFP signals and resulted in two peaks that showed different GFP signals in some cases. On the other hand, the population of yeast cells expressing the split-GFP probes from the genomic DNA was detected as a single sharp peak, indicating the low variations in the GFP signals. With the plasmid-based method, it has been impossible to judge whether no or very weak GFP signals are simply due to the low expression levels of the split-GFP proteins or abnormal organelle contacts because the variations in the expression levels of the split-GFP proteins in each cell are large. The genome-based split-GFP system, however, made it possible to count all yeast cells for quantification because we can detect similar levels of GFP signals with less variability in almost all yeast cells, making quantification of organelle-organelle contacts much easier.

**FIGURE 4:**
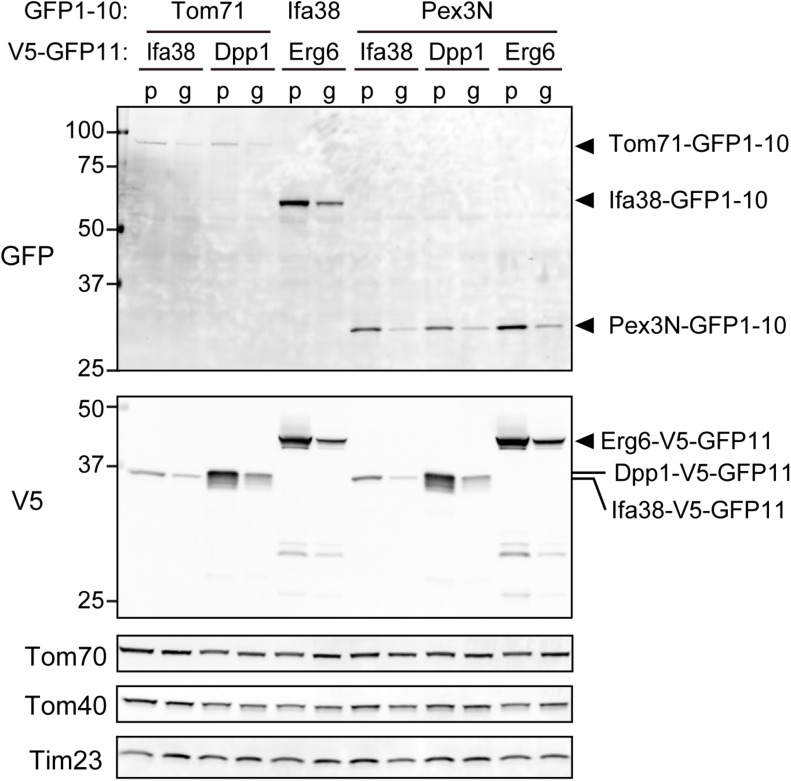
The steady state levels of the split-GFP probes. Immunoblotting of total cell lysates prepared from yeast cells expressing the indicated split-GFP probes from the genomic DNA (g) or plasmid DNAs (p). Monoclonal anti-GFP and anti-V5 antibodies were used to detect the split-GFP proteins. Tom70, Tom40, and Tim23 were used as loading controls.

We next tested if the expression levels of the split-GFP probes were indeed reduced in the present system as compared with those in the previous one. We performed immunoblotting using whole cell extracts prepared from yeast cells expressing the split-GFP probes such as Tom71-, Ifa38-, or Pex3N-GFP1-10 together with Ifa38-, Dpp1-, or Erg6-V5-GFP11 from the plasmids or genomic DNA. The results clearly showed that all the split proteins were expressed at lower levels when expressed from the genomic DNA (Figure 4, g) as compared those in the previous one (Figure 4, p). The overall expression levels were decreased to 30–50%. These results suggest that the genome-based split-GFP system in yeast is advantageous in terms of minimizing artificial organelle-contact sites, consequently improving the quantification of organelle-organelle interactions.

### Validation of the Chromosomally Expressed Split-GFP Probes in Yeast Cells

**FIGURE 5:**
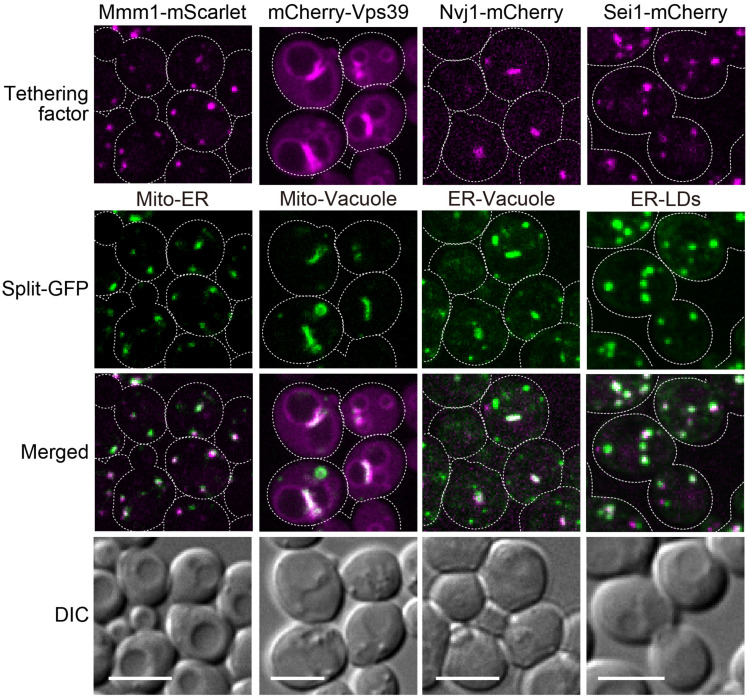
Co-localizations of reconstituted split-GFP probes with ERMES, NVJ, vCLAMP, and ER-LD contact sites. Yeast cells expressing the split-GFP probes on mitochondria and the ER (Mito-ER), the MOM and vacuole (Mito-Vacuole), the ER and vacuole (ER-Vacuole), or the ER and LDs (ER-LDs) were imaged by a fluorescence confocal microscope. To visualize ERMES, NVJ and ER-LD contact sites, Mmm1-mScarlet, Nvj1-mCherry, and Sei1-mCherry were chromosomally expressed, respectively. mCherry-Vps39 was expressed under the *ADH1* promoter from a *CEN-URA3* plasmid to visualize vCLAMP regions. For MOM-ER, ER-vacuole, and ER-LD pairs, maximum projection images were shown. For the mitochondria-vacuole pair, a single focal plane was shown. Scale bars, 5 μm.

We previously confirmed that the plasmid-based split-GFP system could visualize the ER-mitochondria contact sites using the ERMES complex labeled with RFP as a reference (Kakimoto et al., 2018). We thus examined if the genome-based system also properly worked as organelle contact site markers. For this purpose, we visualized the ER-mitochondria, nucleus-vacuole, mitochondria-vacuole, and ER-LDs contact sites by expressing red-fluorescent protein fused to known organelle tethering factors such as Mmm1, Nvj1, Vps39, and Sei1 (Pan et al., 2000; Kornmann et al., 2009; Elbaz-Alon et al., 2014; Hönscher et al., 2014; Grippa et al., 2015). Consistent with our previous study, the GFP signals arising from assembled Tom71-GFP1-10 and Ifa38-V5-GFP11 (MOM-ER), Ifa38-GFP1-10 and Dpp1-V5-GFP11 (ER-vacuole), Tom71-GFP1-10 and Dpp1-V5-GFP11 (MOM-vacuole), or Ifa38-GFP1-10 and Erg6-V5-GFP11 (ER-LDs) were well co-localized with Mmm1-mScarlet, Nvj1-mCherry, mCherry-Vps39, or Sei1-mCherry signals, which represent the ER-mitochondria, nuclear-vacuole, mitochondria-vacuole, or ER-LDs contact sites, respectively (Figure 5). Although Nvj1-mCherry exclusively stained the nuclear-vacuole contact sites (NVJ), the split-GFP signals arising from the ER-vacuole pair showed not only the NVJ signals but also granular GFP signals, which did not correspond to the NVJ regions. This is consistent with our previous observation and suggests the presence of contact sites between the peripheral ER and vacuole (Kakimoto et al., 2018).

### Observations of Organelle Contact Sites in the Absence of the Known Organelle Tethering Factors

**FIGURE 6:**
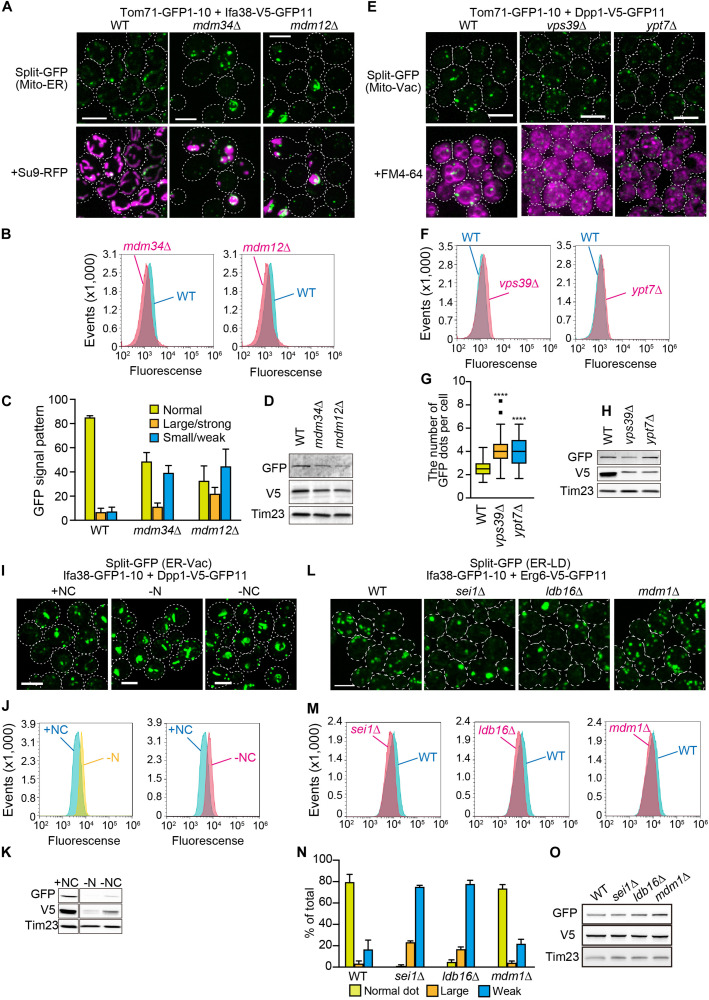
Effects of the lack of known organelle-tethering factors on the organelle-organelle interactions. Logarithmically growing yeast cells expressing the split-GFP probes on **(A)** the ER and MOM (ER-Mito), **(E)** the MOM and vacuole (Mito-Vac), **(I)** the ER and vacuole (ER-Vac), or **(L)** the ER and LDs (ER-LD) were imaged by a fluorescence confocal microscope. To visualize mitochondria and the ER, we expressed Su9-RFP and BipN-mCherry-HDEL, respectively from plasmid DNAs. **(I)** For starvation experiments, yeast cells cultured in the SCD medium were further incubated in the SD-N or S-NC medium for 9 h, and then imaged. **(B,F,J,M)** Yeast cells were analyzed by FACS. **(C,N)** GFP signal patterns or **(G)** the number of GFP dots per cell were quantified. **(D,H,K,O)** Immunoblotting of whole cells extracts prepared from the indicated cells were performed. We detected the split-GFP proteins using anti-V5 and anti-GFP antibodies. Tim23 was used as a loading control. We performed three independent experiments for each condition. Error bars represent standard errors of three independent experiments. We counted total 223, 336, and 208 cells of wild-type, *mdm34Δ*, and *mdm12Δ* cells, respectively **(C)**, and total 217, 285, 259, or 389 cells of wild-type, *sei1Δ*, *ldb16Δ*, and *mdm1Δ* cells, respectively **(N)**. ****p < 0.0001. All images were maximum projections. Scale bars, 5 μm.

We next examined if the genome-based split-GFP system worked as quantitative indicators of dynamic changes in organelle contact sites. Firstly, we visualized the ER-mitochondria contact sites with Tom71-GFP1-10 and Ifa38-V5-GFP11 in the absence of Mdm34 and Mdm12, which are core subunits of the ERMES complex. To quantify the GFP signals that would reflect the ER-mitochondria contacts, we took advantage of FACS analysis. Intriguingly, we could detect *mdm34*Δ or *mdm12*Δ cells as a single peak that showed smaller GFP signals distinct from the one of wild-type cells (Figure 6B), suggesting that the formation of ER-mitochondrial contacts was impaired in the absence of Mdm34 or Mdm12. On the other hand, we noticed that the small populations of *mdm34*Δ and *mdm12*Δ cells exhibited stronger GFP signals than wild-type cells (Figure 6B). This suggests that the formation of ER-mitochondria contacts is restored or enhanced in a fraction of *mdm34*Δ and *mdm12*Δ cells. Consistently, our microscopic analyses revealed that ∼10 or 20% of *mdm34*Δ or *mdm12*Δ cells showed large strong GFP signals whereas ∼40% of them exhibited no or small granular GFP signals (Figures 6A,C). These results suggest that although the ERMES complex is critical for the contact formation, alternative factors could compensate when the ERMES complex is inactive. The diminished GFP signals could be due to the decreases in the steady state levels of the split-GFP proteins (Figure 6D). However, we noticed that the decreased split-GFP protein levels did not necessarily diminish GFP signals. For example, when we observed the mitochondria-vacuole contact sites by expressing Tom71-GFP1-10 and Dpp1-V5-GFP11 from the genomic DNA in yeast cells lacking Vps39 or Ypt7, which are the vCLAMP components (Elbaz-Alon et al., 2014; Hönscher et al., 2014; González Montoro et al., 2018), we did not see decreases in the GFP signals and rather observed the slightly increased signals although the Dpp1-V5-GFP11 levels were decreased (Figures 6E,F,H). Our microscopic analyses showed that the number of GFP dots was increased in the absence of Vps39 or Ypt7 (Figure 6G). These results suggest that Vps39 and Ypt7 are not essential for the formation of mitochondria-vacuole contact sites.

Previous studies reported that NVJ regions expanded when yeast cells were subjected to starvation (Kvam and Goldfarb, 2006; Toulmay and Prinz, 2012; Hariri et al., 2018). Interestingly, despite the drastic decreases in the amounts of split-GFP proteins, Ifa38-GFP1-10 and Dpp1-V5-GFP11 under the nitrogen and carbon starvation conditions (Figure 6K), we observed clear increases in the GFP signals showing the ER-vacuole contact sites including NVJs (Figures 6I,J). These results indicate that the split-GFP system has the extra capacity to sense an increase in inter-organelle contacts even when the amount of split-GFP proteins is reduced.

To further validate the potency of this split-GFP system in quantifying organelle-organelle interactions, we visualized ER-LD contact sites by expressing Ifa38-GFP1-10 and Dpp1-V5-GFP11 in various mutant cells lacking Sei1, Ldb16, or Mdm1, which were reported to tether these two organelles (Wolinski et al., 2011; Grippa et al., 2015; Henne et al., 2015; Hariri et al., 2019). Strikingly, our FACS analyses showed that *sei1*Δ and *ldb16*Δ cells were sorted as distinct peaks that showed smaller GFP signals as compared with the one wild-type cells (Figure 6M). Consistent with the FACS results, we observed that the approximately 80% of *sei1*Δ and *ldb16*Δ cells showed the small and/or dim GFP signals while 90% of wild-type cells showed clear granular GFP signals that correspond to Sei1-mCherry (Figures 5, 6L,N). On the other hand, *mdm1Δ* cells exhibited weaker GFP signals than that of wild-type cells (Figure 6M), although the GFP signal patterns looked quite similar to that of wild-type (Figure 6N), suggesting that Mdm1 plays a minor role in the ER-LD contact formation. We confirmed that the steady state levels of the split-GFP proteins were comparable among yeast cells tested here (Figure 6O). These observations consistently support the previous finding that the seipin complex Sei1/Ldb16 stabilizes ER-LD contact sites (Grippa et al., 2015). In summary, we conclude that the genome-base split-GFP system is useful tool to assess dynamic changes in the degree of organelle-organelle contacts.

### The Inducible Split-GFP System in HeLa Cells

**FIGURE 7:**
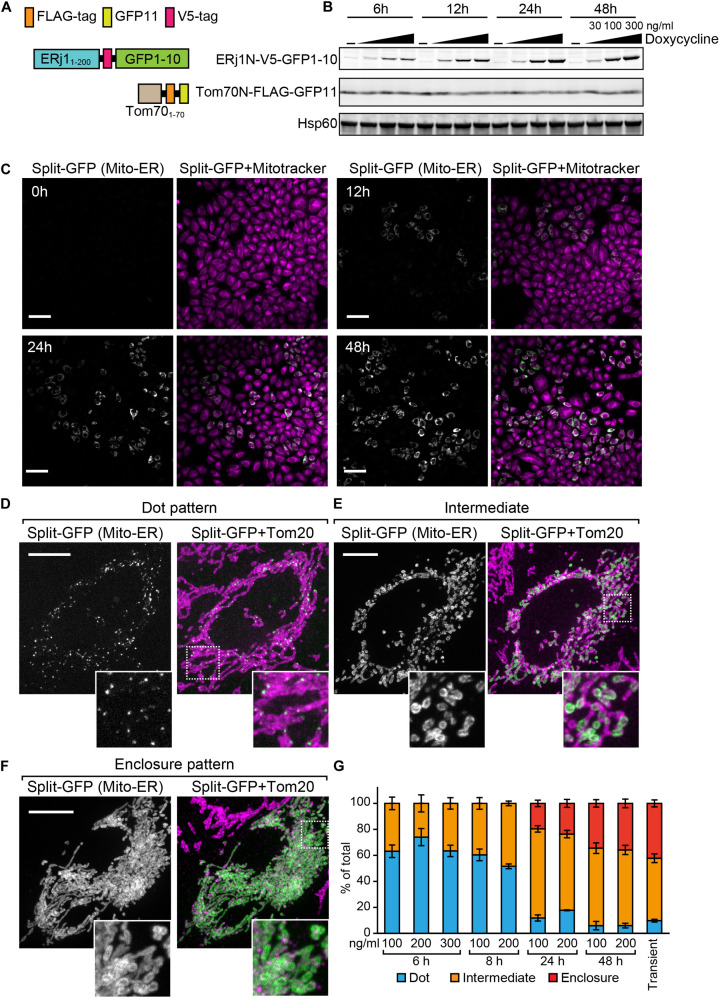
Characterization of the HeLa cell line that enables inducible expression of the split-GFP probes in the presence of doxycycline. **(A)** Schematics of the split-GFP probes used in the HeLa cell line. **(B)** Western blotting of whole cell extracts prepared from the HeLa cells cultivated with different concentration (0, 30, 100, and 300 ng/ml) of doxycycline for the indicated time. Scale bars represent 100 μm. **(C)** The HeLa cells were imaged by fluorescence confocal microscopy 0, 12, 24, and 48 h after the addition of doxycycline. Mitochondria were stained with mitotracker. Scale bars, 10 μm. **(D,E)** Representative images of the HeLa cells after 6 h-induction of ERj1N-V5-GFP11 with 200 ng/ml doxycycline, or **(F)** transiently expressing the same probes were fixed and then imaged by a confocal fluorescence microscope. Mitochondria were stained by anti-Tom20 antibodies. Maximum projection images were shown. Scale bars represent 10 μm. **(G)** We performed 3 independent experiments and counted more than 60 cells showing GFP signals in total. Error bars represent standard errors (*n* = 3).

Previously, we transiently expressed the split-GFP probes on the ER and MOM to detect the ER-mitochondria-contact sites in HeLa cells (Kakimoto et al., 2018). However, in this transient method, it was difficult to control the expression levels of the split-GFP proteins (Figures 1E,F). We thus aimed to develop a more reliable split-GFP system in which the expression of the split-GFP probes could be controlled in HeLa cells. To achieve this, we utilized the Tet-One inducible expression system (Takara Bio USA, Inc.). In this system, we are able to induce the expression of a gene of interest, which is located downstream of the tetracycline-response element (TRE), by activating a transcription activator named rtTA with doxycycline (Gossen et al., 1995). First, we constructed a stable HeLa cell line expressing the MOM-targeted split-GFP protein Tom70N-FLAG-GFP11, comprising the first 70 N-terminal residues of Tomm70 followed by a 3xFLAG tag and GFP11 (Figure 7A). Subsequently, we cloned the gene encoding another split-GFP protein, ERj1N-V5-GFP1-10, which comprises the first 200 N-terminal residues of ERj1 followed by a V5 tag and GFP1-10, into the multi-cloning site downstream of the TRE of a pTet-One vector, which also expresses rtTA (Figure 7A). We then transfected the plasmid with a hygromycin marker into HeLa cells. To check the inducible expression of ERj1N-V5-GFP1-10, we prepared whole cell lysates from the resulting HeLa cells, which were cultured in the presence or absence of different concentrations of doxycycline for different periods of time. Immunoblotting using the whole cell extracts showed that Tom70N-FLAG-GFP11 was stably expressed, while the expression of ERj1N-V5-GFP1-10 was induced by adding doxycycline. In particular, its expression was initially observed 6 h after the addition of doxycycline, and it was found that increased drug concentration or prolonged incubation time led to higher expression levels (Figure 7B). As the expression of the split-GFP protein was induced, we observed an increase in GFP signals (Figure 7C). These results indicated that we were able to control the expression levels of the split-GFP probes, which enabled us to minimize the undesired secondary effects caused by the excess reconstitution of the probes (Figure 1F). We further aimed to optimize the appropriate conditions required to observe small, dot-like GFP signals for the ER-mitochondria contact sites in HeLa cells. To this end, we tested various conditions with different concentrations of doxycycline and incubation time periods. Whereas the prolonged cultivation with doxycycline resulted in clear GFP signals (Figure 7C), relatively short-time induction (6 h) with 200 ng/ml doxycycline was the best condition to obtain dot-like GFP signals (Figure 7D). At the optimal condition, ∼70% of HeLa cells contained dot-like GFP signals (Figure 7D), while ∼30% of the cells showed ring-like GFP signals, which surrounded a part of mitochondria (Figure 7E, Intermediate pattern). The prolonged incubation led to an increase in the ratio of the HeLa cells showing the intermediate pattern as well as GFP signals that enclosed entire mitochondria (Figure 7F, Enclosure pattern). The previous transient expression system was comparable to the condition of the present inducible system with long cultivation (48 h) (Figure 7G). These results suggest that the present system offers a more accurate view of the ER-mitochondria contact sites as compared with the previous transient system.

### Live Cell Imaging of HeLa Cells Expressing the Inducible Split-GFP Probes

**FIGURE 8:**
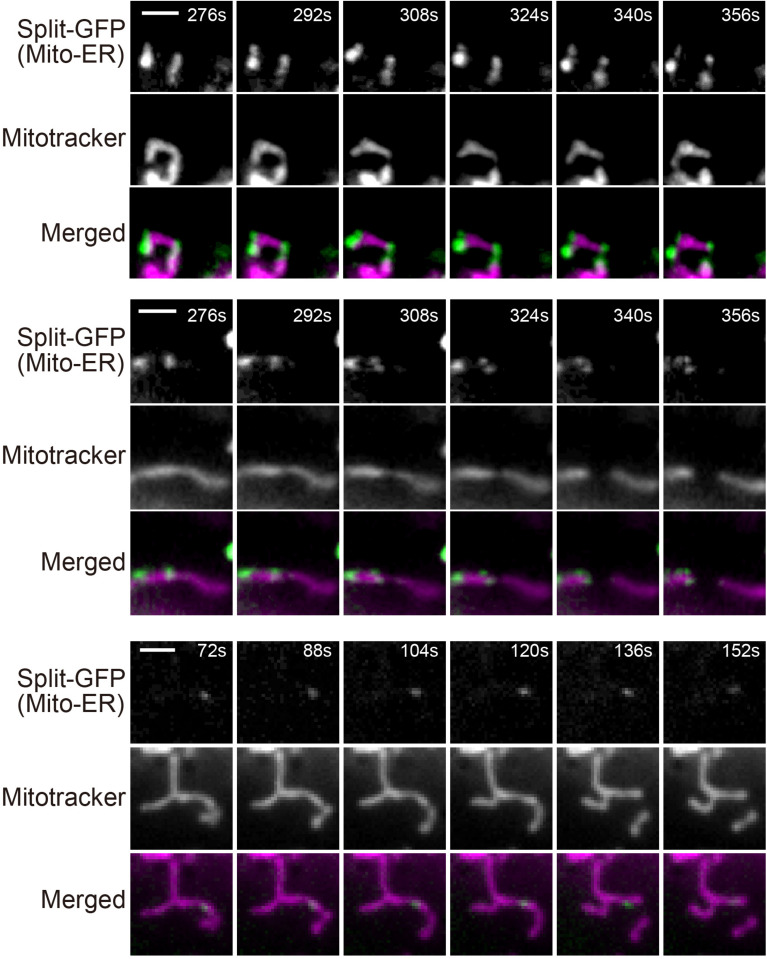
Live-cell imaging of HeLa cells expressing the split-GFP probes on the ER and MOM. The HeLa cells were cultivated in the presence of 300 ng/ml doxycycline for 8 h and imaged by fluorescence microscopy. Mitochondria were stained with mitotracker. Three mitochondrial division events observed in Supplementary Movies 1,2, were shown. Scale bars, 2 μm.

Previous studies reported that ER tubules wrap around and constrict mitochondria to determine the sites of mitochondrial division (Friedman et al., 2011; Lewis et al., 2016). We therefore examined if mitochondrial fission occurs where granular GFP signals arising from the split-GFP proteins were present to confirm that the split-GFP probes really mark the ER-mitochondria contact sites in HeLa cells. To this end, we performed live-cell imaging of the HeLa cell after inducing the expression of ERj1N-V5-GFP1-10 and acquired time-lapse images every 4 s. The reconstituted time-lapse movies showed that mitochondrial fission occurred at or next to the sites that the GFP signal existed (Supplementary Movies 1,2 and Figure 8). These results suggest that the complete GFP molecules are reconstituted near authentic ER-mitochondria contact sites.

## Discussion

Previous studies have shown that split-fluorescent proteins are effective in visualizing inter-organelle-contact sites (Cieri et al., 2018; Kakimoto et al., 2018; Shai et al., 2018; Yang et al., 2018). However, a drawback of this method is the irreversible association of the split-fluorescent proteins, which induces artificial contact sites between different organelles and leads to an abnormality in the structure and function of the organelles (Figure 1F; Bishop et al., 2019). In this study, we found that the incorporation of the split-GFP genes into the yeast genome resulted in a decrease in the expression levels of the split-GFP proteins (Figure 4). Noteworthy, this decrease in expression levels successfully diminished the formation of unnecessary inter-organellar contacts (Figure 3). In addition, the variation in its expression levels among cells also decreased dramatically, which is advantageous in improving the quantitative evaluation of the organelle-contact sites. In fact, with the genome-based split-GFP system, we were able to detect changes in the organelle contacts, which had been difficult to judge by using the previous method. Specifically, we could observe clear differences in the GFP signal intensities and patterns when known organelle-tethering factors were absent (Figure 6).

Previously, a high-throughput microscopy system that makes use of the Yeast Knock-out deletion collection expressing the split-Venus probe successfully identified the tethering factors between the mitochondria and peroxisomes (Shai et al., 2018). However, such a large-scale microscope system is not commonly available and cannot be easily set up. With our improved split-GFP system, genetic screening experiments to search for factors involved in organelle-organelle contacts may be performed more easily. As mentioned above, our new split-GFP system resulted in lower GFP signals with small variation (Figure 3G), so that yeast cells expressing the split-GFP probes could be detected as a single sharp peak by flow cytometry using GFP fluorescence as an index (Figure 3H). It should be noted that almost all wild-type yeast cells showed the similar GFP signals when the split-GFP probes were expressed from the genomic DNA. This feature is advantageous for high-content screening by FACS as compared with the previous plasmid-based system in which yeast cells were detected as two wider peaks (Figure 3H). Therefore, this allows us to use fluorescence-activated cell sorting (FACS) to easily separate the subpopulation of cells in which the GFP signals are either diminished or enhanced after introducing random mutations or the yeast genome library cloned in a high copy number vector for the gene overexpression. This simple FACS study may help to perform comprehensive searches for the factors involved in inter-organelle-contact sites.

Genetic studies using mammalian cells may be performed by using the siRNA libraries and the CRISPR-Cas9 sgRNA libraries (Shalem et al., 2014). By using the HeLa cell line constructed in this study, it is possible to easily adjust the timing of the split-GFP expression. Expressing the split-GFP probes after introducing a siRNA or a CRISPR-Cas9 sgRNA library allows for a screening protocol wherein the secondary effects of split-GFP association are minimized. The application of this new split-GFP system to the study of various inter-organelle contact sites may lead to the discovery of novel factors involved in inter-organelle interactions in the future.

## Data Availability Statement

The raw data supporting the conclusions of this article will be made available by the authors, without undue reservation.

## Author Contributions

ST and YT designed the research and wrote the manuscript. ST performed experiments using HeLa cells. MS, YK, SF, and YT performed yeast experiments. All authors contributed to the article and approved the submitted version.

## Conflict of Interest

The authors declare that the research was conducted in the absence of any commercial or financial relationships that could be construed as a potential conflict of interest.

## Footnotes

1 http://n2t.net/addgene:62383
